# Premarket Safety and Efficacy Studies for ADHD Medications in Children

**DOI:** 10.1371/journal.pone.0102249

**Published:** 2014-07-09

**Authors:** Florence T. Bourgeois, Jeong Min Kim, Kenneth D. Mandl

**Affiliations:** 1 Division of Emergency Medicine, Boston Children’s Hospital, Boston, Massachusetts, United States of America; 2 Department of Pediatrics, Harvard Medical School, Boston, Boston, Massachusetts, United States of America; 3 Children’s Hospital Informatics Program at the Harvard-MIT Division of Health Sciences and Technology, Boston Children’s Hospital, Boston, Massachusetts, United States of America; 4 Faculty of Arts and Sciences, Wellesley College, Wellesley, Massachusetts, United States of America; 5 Harvard Medical School Center for Biomedical Informatics, Boston, Massachusetts, United States of America; University of Chicago, United States of America

## Abstract

**Background:**

Attention-deficit hyperactivity disorder (ADHD) is a chronic condition and pharmacotherapy is the mainstay of treatment, with a variety of ADHD medications available to patients. However, it is unclear to what extent the long-term safety and efficacy of ADHD drugs have been evaluated prior to their market authorization. We aimed to quantify the number of participants studied and their length of exposure in ADHD drug trials prior to marketing.

**Methods:**

We identified all ADHD medications approved by the Food and Drug Administration (FDA) and extracted data on clinical trials performed by the sponsor and used by the FDA to evaluate the drug’s clinical efficacy and safety. For each ADHD medication, we measured the total number of participants studied and the length of participant exposure and identified any FDA requests for post-marketing trials.

**Results:**

A total of 32 clinical trials were conducted for the approval of 20 ADHD drugs. The median number of participants studied per drug was 75 (IQR 0, 419). Eleven drugs (55%) were approved after <100 participants were studied and 14 (70%) after <300 participants. The median trial length prior to approval was 4 weeks (IQR 2, 9), with 5 (38%) drugs approved after participants were studied <4 weeks and 10 (77%) after <6 months. Six drugs were approved with requests for specific additional post-marketing trials, of which 2 were performed.

**Conclusions:**

Clinical trials conducted for the approval of many ADHD drugs have not been designed to assess rare adverse events or long-term safety and efficacy. While post-marketing studies can fill in some of the gaps, better assurance is needed that the proper trials are conducted either before or after a new medication is approved.

## Introduction

As many as 10% of children and adolescents in the United States carry a diagnosis of attention-deficit hyperactivity disorder (ADHD), making it one of the most common conditions of childhood. [1], [2] The single most efficacious treatment for reducing symptoms of ADHD are medications and most children diagnosed with ADHD receive pharmacotherapy. [1], [3], [4] Clinicians and patients have a large therapeutic armamentarium to choose from, with over a dozen ADHD medications marketed in a wide array of formulations and delivery systems [5].

The short-term therapeutic response to ADHD medications is upwards of 70%, among the highest of psychiatric medications. [6], [7] As a result, although ADHD typically requires long-term treatment, the short-term efficacy of ADHD drugs can be assessed after a brief treatment period, often on the order of days to weeks [6].

Since most trials focus on efficacy measures as primary endpoints, clinical trials testing ADHD medications may be relatively short, resulting in drugs with limited long-term safety or efficacy data. [8] Further, since studies are powered to measure efficacy endpoints, sample sizes may be inadequate to detect rare adverse events related to ADHD medications [7].

Guidelines for the safety and efficacy evaluation of drugs are provided by the International Conference on Harmonization of Technical Requirements for Registration of Pharmaceuticals for Human Use (ICH). [9] Though not binding, these requirements guide clinical trial practice by the United States Food and Drug Administration (FDA). [10] The ICH provides specific recommendations on the safety assessment of chronic drugs used in the treatment of non-life-threatening conditions, which includes ADHD. [11] The guideline states that since most adverse drug events first occur and are most frequent during the initial months of treatment, approximately 300 to 600 patients should be treated for at least 6 months prior to market availability. Some adverse events may increase in frequency or severity over time or may only occur after several months of treatment; therefore, at least 100 patients should be exposed to a drug for at least 12 months. And finally, the guidelines recommend that the total number of patients treated with a medicine prior to approval, including short-term exposure, should be about 1500.

To assess the extent to which rare adverse events and the long-term safety and efficacy of ADHD drugs have been evaluated prior to their market authorization, we sought to identify and evaluate all safety and efficacy trials submitted to the FDA for the approval of ADHD drugs and quantify the number of participants studied and their length of exposure prior to the market availability of each ADHD medicine. We hypothesized that the premarket study of many ADHD medications in children has been insufficient to adequately assess rare adverse events and long-term safety and efficacy.

## Methods

### Drugs Used in the Treatment of ADHD

We identified all drugs approved by the FDA for ADHD using information available on the FDA website as well as other online resources. [12], [13] All products with a unique NDA number were selected. We recorded the drugs’ FDA approval date for the indication of ADHD, the approved ages for treatment, the drug formulation, the pharmaceutical company sponsoring the application, and the current marketing status of the drug. When there was no FDA approval date for ADHD, we used the date of the first label on which ADHD was listed as an indication for the drug (N = 2).

### Data Sources and Extraction

For each drug identified, we obtained the FDA Drug Approval Package, which includes the medical and statistical reviews, approval letters, drug labels, and any related administrative documents and correspondence. For newer agents, these documents are posted on the FDA website. [13] When the approval packages were not available, they were obtained from the FDA through a Freedom of Information Act request.

We reviewed the approval documents and identified the clinical trials performed by the sponsor and used by the FDA to evaluate the drug’s clinical efficacy or safety in the review process. If the agent was not a New Molecular Entity (and thus prior trials and FDA review had been performed), we reviewed the clinical trials in the original FDA approval package in order to identify any pediatric patients who may have been studied in safety or efficacy assessments. Thus, for drugs representing new formulations or drugs repurposed for the indication of ADHD and for which the FDA approval process might be modified and possibly less stringent given the prior review, we analyzed all clinical trials that had been conducted for approval at any stage. Several drugs had FDA approvals that either recommended or required the conduct of additional, post-marketing clinical trials. When the approval packages did not include supplemental information on these trials, we searched the online trial registry ClinicalTrials.gov to determine whether additional trials had been performed. We also contacted the pharmaceutical companies who had sponsored the drug applications to inquire about clinical trials performed in response to the FDA requests.

For each medication we extracted the following information: number of clinical trials supporting the application, number of participants studied who received at least one dose of the study drug, participant age ranges, trial duration at dosage levels intended for clinical use (i.e. excluding open-label titration periods), and comparators (if any) employed. Trials were categorized as efficacy or safety trials, with safety trials defined as those explicitly labeled as safety by the sponsor.

The clinical trials related to the drug approvals were linked to publications in the medical literature with a search of PubMed. Trials and publications were matched on the drug name, trial design characteristics, number of participants, and trial duration [14].

### Data Analysis

Descriptive statistics were used to report the total number of participants studied for less than 6 months, for at least 6 months, and for at least 12 months. Since many drugs used in the treatment of ADHD have been on the market for many years and the FDA approval process may have evolved over this period, we also calculated these values for the drugs approved in the last 10 years (since 2004).

## Results

We identified 20 drugs approved by the FDA via a New Drug Application (NDA) for the treatment of ADHD in children (Table 1). These drugs represent 10 different active ingredients with the individual products differing based on the formulation and delivery systems. Most drugs were approved for children 6 years and older with two drugs approved for children as young as 3 years old. Only 3 of the drugs have been discontinued. Original drug approval packages were not available on the FDA website and therefore obtained via a Freedom of Information Act (FOIA) request for Ritalin, Ritalin SR, Desoxyn, Cylert, Dexedrine, Biphetamine, Adderall, and Adderall XR.

**Table 1:** Medications approved by the FDA for the treatment of ADHD.

Active ingredient	Brand name^a^	FDA approvaldate^b^	Approvedages foruse	Formulationand deliverysystem	Pharmaceutical companysponsoring application	Marketing status
***Stimulants***						
Methylphenidatehydrochloride	Ritalin	12/5/1955	6 years andolder	Oral tablet	Novartis	Prescription
	Ritalin SR	3/30/1982	6 years andolder	Oral extended-releasetablet	Novartis	Prescription
	Concerta	8/1/2000	6 years andolder	Oral extended-releasetablet, OROS	Janssen Pharms	Prescription
	Metadate CD	4/3/2001	6 years andolder	Oral extended-releasecapsule	UCB Inc.	Prescription
	Ritalin LA	6/5/2002	6 years andolder	Oral extended-releasecapsule, SODAS	Novartis	Prescription
	Methylin	12/19/2002 4/15/2003	6 years andolder	Oral solution Oralchewable tablet	Mallinckrodt	Prescription
	Daytrana	4/6/2006	6 years andolder	Transdermalextended-release film	Noven Pharms	Prescription
	Quillivant XR	9/27/2012	6 to 17years	Oral extended-releasesuspension	Nextwave Pharms	Prescription
Methamphetaminehydrochloride	Desoxyn	1965^c^	12 years andolder	Oral tablet	Lundbeck	Prescription
Pemoline	Cylert	1/27/1975 1/30/1976	6 years andolder	Oral tablet Oralchewable tablet	Abbott	Discontinued
Dextroamphetaminesulfate	Dexedrine	8/2/1976	3 years andolder	Oral extended-releasecapsule	Amedra Pharms	Prescription
Amphetamine,dextroamphetaminemixed salts	Biphetamine	1979^c^	3 years andolder	Oral extended-releasecapsule	UCB Inc.	Discontinued
	Adderall	02/13/1996	3 years andolder	Oral tablet	Teva Womens	Discontinued
	Adderall XR	10/11/2001	6 years andolder	Oral extended-releasecapsule	Shire	Prescription
Dexmethylphenidatehydrochloride	Focalin	11/13/2001	6 years andolder	Oral Tablet	Novartis	Prescription
	Focalin XR	5/26/2005	6 years andolder	Oral extended-releasecapsule, SODAS	Novartis	Prescription
Lisdexamfetaminedimesylate	Vyvanse	2/23/2007	6 to 12years	Oral capsule	Shire	Prescription
***Non-stimulants***						
Atomoxetinehydrochloride	Strattera	11/26/2002	6 years andolder	Oral capsule	Eli Lilly	Prescription
Guanfacinehydrochloride	Intuniv	9/2/2009	6 years andolder	Oral extended-releasetablet	Shire	Prescription
Clonidinehydrochloride	Kapvay	9/28/2010	6 years andolder	Oral extended-releasetablet	Shionogi Inc.	Prescription

a All drugs approved by the FDA under a New Drug Application (NDA).

b Approval date for treatment of ADHD.

c Date derived from first product labeling to include ADHD as an indication as the FDA approval package for biphetamine and desoxyn do not include ADHD as one of the drug indications.

### Characteristics of Clinical Trials

There were 32 clinical trials conducted by sponsors prior to the initial FDA approval of the ADHD drugs. Of these, 5 studies–representing 3 drugs–were safety studies. The minimum age of participants in all of the trials was 6 years. Six of the drugs were studied in efficacy trials that included an active comparator (5 included both an active comparator and placebo) while all others were approved based on comparison to placebo alone. Among the 10 most recently approved drugs, only 2 included comparison to one of the ADHD drugs already marketed. A total of 8 (25%) of the trials have been published in the medical literature.

### Number of Participants and Length of Study

The median number of participants studied per drug was 75 (IQR 0, 419; range 0 to 660). Overall, 2756 (66%) participants were studied for less than 6 months, 746 (18%) for 6 to 11 months, and 688 (16%) for 12 or more months (Table 2).

**Table 2:** Clinical trials conducted for the FDA approval of ADHD drugs in children.

Brand name	Number and type of clinical trials	Participant age ranges	Number of participants studied and treated	Duration of trial and follow up	Comparator(s)	Trials published
Desoxyn	0					
Ritalin	0					
Ritalin SR	3 efficacy	unknown	48^a^	2 weeks	Ritalin	0
Concerta	3 efficacy	6 to 12 years	64	1 week	Placebo/Ritalin	0
			70	1 week		1[30]
			94	4 weeks		1[42]
	1 safety	6 to 13 years	432^b^	6 months	None	1[43]
Metadate CD	1 efficacy	6 years and older	158	3 weeks	Placebo	0
Ritalin LA	2 efficacy	6 to 12 years	34	2 weeks	Placebo	0
			63			
Methylin	0^c^					
Daytrana	2 efficacy	6 to 12 years	79	1 week	Placebo	0
			96	2 weeks	Placebo/Concerta	1[44]
	2 safety		127^b^	6 months	None	0
			327^b^	1 year		
Quillivant XR	1 efficacy	6 to 12 years	45	2 weeks	Placebo	0
Cylert	0^d^					
Dexedrine	0^e^					
Biphetamine	0^e^					
Adderall	0^f^					
Adderall XR	2 efficacy	6 to 12 years	360	3 weeks	Placebo	0
			51	1 week	Placebo/Adderall	
Focalin	2 efficacy	6 to 17 years	43	4 weeks	Placebo/Methylphenidate	0
			35	20 weeks	Placebo	
	2 safety		187^b^	6 months	None	0
			361^b^	1 year		
Focalin XR	1 efficacy	6 to 17 years	53	7 weeks	Placebo	0
Vyvanse	2 efficacy	6 to 12 years	50	1 week	Placebo/Adderall	1[45]
			213	4 weeks	Placebo	1[46]
Strattera	4 efficacy	6 years and older	213	8 weeks	Placebo	0
			85	6 weeks		
			65	9 weeks		
			64	9 weeks		
Intuniv	2 efficacy	6 to 17 years	259	8 weeks	Placebo	1[47]
			258	9 weeks		0
Kapvay	2 efficacy	6 to 17 years	154	8 weeks	Placebo	1[48]
			102			0

a Assumed equal distribution of randomized participants between the two study arms.

b Not all unique patients as some participants from efficacy trial subsequently enrolled in safety trial.

c Methylin was a new formulation of FDA-approved methylphenidate.

d FDA approval package includes information on pre-clinical trials only and there is no mention of the conduct of clinical trials.

e The FDA approval package included clinical trials of Dexedrine and Biphetamine for the treatment of obesity in adults, but no clinical trials assessing their use for the treatment of ADHD were identified.

f Adderall was originally approved in 1960 as an anorectic under the brand name Obetrol, but no clinical trials assessing their use for the treatment of ADHD were identified.

Seven drugs were approved by the FDA for the treatment of ADHD without the submission of any clinical trial data by the sponsors. Of these, 5 were approved between 1943 and 1975, one (Adderall) in 1996 and another (Methylin) in 2003. Ritalin, the oldest drug approved for ADHD, was approved based on prior clinical experience, which was presented as a bibliography. Methylin was a new formulation of previously approved methylphenidate that underwent an NDA but without the submission of pediatric efficacy trials. [15] Desoxyn was originally approved in 1943 as an anorectic in the treatment of obesity but no clinical trials assessing its safety in children or its efficacy in the treatment of ADHD in children were identified in the original approval package. The indication for ADHD is first included on the drug label in 1965 without supporting clinical trials in the accompanying documentation. The approval package for Cylert includes information on pre-clinical trials without mention of clinical trials conducted for any indication. Dexedrine was approved for the treatment of narcolepsy, obesity, and ADHD, but the approval package does not include trials enrolling children or studying efficacy for ADHD. Similarly, Biphetamine was previously approved as an anorectic in adults, but the original approval package does not include safety assessments in children and ADHD was added as an indication in product labels starting in 1979 without supporting clinical trials. Adderall was originally approved in 1960 as an anorectic under the trade name Obetrol before being marketed without FDA approval as Adderall for the treatment of ADHD. Approval was subsequently obtained without clinical trials assessing its safety in children or efficacy in ADHD [16].

Among the 13 drugs with clinical trials studying ADHD, a total of 4 drugs (31%) were approved after less than 100 participants had been studied while 7 drugs (54%) were approved after study in less than 300 participants. Efficacy trials ranged from 1 to 10 weeks and safety trials from 6 to 12 months in length. The median trial length prior to approval was 4 weeks (IQR 2, 9; range 1 to 52 weeks), with 5 (38%) drugs approved after participants were studied for less than 4 weeks, 7 (54%) after less than 8 weeks, and 10 (77%) after less than 6 months.

Focusing on the 6 drugs approved since 2004, the median number of participants studied per drug was 259 (IQR 53, 517; range 45 to 629) and there were 1309 (74%), 127 (7%), and 327 (18%), participants studied for less than 6 months, 6 to 11 months, and 12 or more months of study, respectively. The median trial length prior to approval was 8 weeks (IQR 2, 20; range 1 to 52 weeks).

Based on the ICH guidelines for safety assessments of chronic medications, none of the drugs met the recommendation for 1500 participants studied prior to drug approval (Table 3). Three of the drugs, Daytrana, Focalin, and Concerta (15% of all ADHD drugs), met requirements to have at least 300 participants total exposed to the drug for 6 months prior to marketing. Daytrana and Focalin (10% of all ADHD drugs) were also compliant with the recommendation to study at least 100 participants for 12 months.

**Table 3:** Compliance of pre-approval ADHD clinical drug trials with ICH guidelines overall and during past 10 years.

Time period	300 participants exposed for at least 6 months, N (%)	100 participants exposed for at least 12 months, N (%)	1500 participants exposed in total
Drugs approved pre-2004 (N = 14)	2 (14)	1 (7)	0
Drugs approved 2004 to 2013 (N = 6)	1 (17)	1 (17)	0
All drugs (N = 20)	3 (15)	2 (10)	0

### Clinical Trials Requested by the FDA

FDA approval of 4 of the drugs included required submission of additional clinical trials by the sponsor and 2 drug approvals included recommendations for additional trials (Table 4). The basis for the required studies cited by the FDA included concerns over limited long-term safety data, limited study in the adolescent population, and insufficient information on specific potential adverse events [17]–[19].

**Table 4:** Clinical trials recommended or required by the FDA at the time of drug approval^a^.

Brand name	Recommendedor required	Details of recommendedor required trials	Clinical trialsconducted	Participant age range	Number of participantsstudied and treated	Duration of trialand follow-up	Trial publications
Ritalin	Recommended	Safety studies of Ritalin when administeredto children for periods of 3, 6, 12, and 24 months,with specific reference to blood counts, including platelets,to possible causes of abdominal pain, cardiovascular function, and to growth	0				
Daytrana	Required	Safety study to investigate and characterize contactsensitization associated withthe use of methylphenidate transdermalsystem, to be completed by April 2008	Double-blind, multi-center,parallel-group, doseoptimization safetyand efficacy trialcompleted in 2008	13 to 17 years	217	7 weeks	1[44]
		Study of Daytrana in the treatment of children 13–17 yearsold with ADHD, to be completed by April 2009	Open-label, extensionsafety study completedin 2009		162	6 months	1[49]
Focalin XR	Recommended	Safety and efficacy study in adolescentsof at least 1 year duration	0^b^				
Vyvanse	Required	Clinical study in adolescents with ADHD, tobe completed by February 2010	0^c^				
Intuniv	Required	Long-term maintenance study of efficacy and safety ofguanfacine as monotherapy in children andadolescents to be completed by September 2012	0				
		Efficacy and safety study of guanfacine inadolescents to be completed by September 2012	0				
		Efficacy and safety study of guanfacine as adjunctive treatmentwith long-acting oral psychostimulants to be completed by June 2010	0				
Kapvay	Required	Longer-term randomized withdrawal maintenance study of efficacyand safety of clonidine hydrochloride extended-release tablets asmonotherapy or, alternatively, as adjunctivetherapy, in children and adolescents, tobe completed by December 2013	Parallel-group, placebo-controlled,withdrawal efficacystudy completed in 2012	6 to 17 years	68^d^	26 weeks	0

a All information on the trials obtained from ClinicalTrials.gov.

b Five trials were conducted by Novartis after the approval of Focalin XR, but all were <1 year in length (longest trial 5 weeks) and none focused on adolescents.

c Three phase 4 trials have been registered in ClinicalTrials.gov by Shire since the FDA approval of Vyvanse, including two trials focusing on adolescents. None are listed as completed (NCT01552915, NCT01552902, NCT01328756).

d Assuming 135 participants were equally distributed between active and placebo arms.

The sponsors for Daytrana and Kapvay conducted the required studies, adhering to the timelines imposed by the FDA for study completion. None of the studies required for Vyvanse or Intuniv or any of the studies recommended for Ritalin or Focalin XR could be identified. The corresponding pharmaceutical companies in each of these cases were contacted and were also not able to provide us with information that these trials had been conducted.

## Discussion

This is the first systematic assessment of patient exposure to ADHD medications prior to their approval and market availability. A total of 17 medications are currently available in the U.S. for the treatment of ADHD in children, 6 of which have been approved by the FDA in the last 10 years. These drugs are intended for chronic use in children, but the median trial length prior to approval is 4 weeks and only 3 of the drugs have been assessed in long-term safety trials. The number of exposed children prior to approval is also small, with 14 drugs approved after study in less than 300 participants. Concerta, Daytrana, and Focalin (15% of all ADHD drugs) are the only drugs that meet the ICH recommendations of at least 300 patients studied for 6 months prior to market availability.

Concerns over the lack of safety data–including adverse events and long-term safety issues–for ADHD drugs have been voiced by a number of entities, including the FDA, the NIH, and the European Committee for Medicinal Products for Human Use. [17], [20], [21] Known adverse effects vary for the different drugs and include growth retardation, decreased appetite, insomnia, and cardiovascular effects. [6], [22]–[24] Post-marketing reports of adverse events prompted the FDA in 2007 to require manufacturers of drugs approved for the treatment of ADHD to develop Medication Guides with detailed information for clinicians and parents on possible adverse effects and precautions that can be taken to avoid them. [25], [26] Package inserts of all stimulant drugs for the treatment of ADHD have also been amended to include warning language around specific risks. [7].

Because ADHD is a chronic condition, the long-term efficacy of ADHD drugs also warrants further consideration. [8], [20] Long-term follow up studies of children treated with ADHD medications have not been designed to adequately assess the long-term benefits of medication therapy compared to behavioral interventions. [27], [28] One prospective 8-year follow-up study of children with ADHD who had been treated for at least 14 months with medication indicated there may be no advantage in academic performance or social functioning in children who received medication compared to those managed with behavioral interventions. [28] Additional long-term studies are needed assessing the impact of ADHD treatments into adolescence and of outcome measures beyond ADHD symptoms that capture effects on long-term functioning.

In order for drugs to become available in a timely fashion, large long-term randomized controlled trials may not always be feasible. Instead, post-marketing observational trials can provide additional information on rare adverse events and better delineate a drug’s long-term safety and efficacy. [29] In the European Union, pharmaceutical companies seeking market authorization for a drug must submit a Risk Management Plan with detailed proposals for post-authorization pharmacovigilence activities and efficacy studies. [30], [31] These management plans are considered continuous, evolving processes and can be modified at the request of regulatory agencies as new information–including safety concerns–become available [32], [33].

In the U.S., the FDA historically relied on studies referred to as “post-marketing commitments” to obtain additional safety and efficacy data after a drug was approved. [34] These commitments were agreed upon by the FDA and the pharmaceutical companies at the time of approval and were often mandated as a condition for approval. However, the FDA lacked any avenues for enforcement, and reports showed that as many as two-thirds of requested studies were never started by the pharmaceutical companies. [35], [36] The FDA Amendments Act of 2007 granted the FDA additional authority to require post-marketing studies–now referred to as “post-marketing requirements”–and also expanded the conditions under which the FDA could make the requests. [37] The FDA may now take enforcement action to ensure studies are conducted and recent reports have shown substantial improvement in compliance with study requests [38].

While we found poor compliance with post-marketing study requests for ADHD drugs, moving forward, these studies may represent an important tool for the detection of rare adverse events and the long-term safety and efficacy of medications. However, observational studies are not equivalent to randomized controlled trials powered to detect adverse events or specific efficacy end-points, nor can they approximate the rigor of randomized trials in accounting for treatment selection bias and other design issues. [39] Instead, post-marketing observational studies should complement pre-approval clinical trials, designed to include sufficient patient exposure to reasonably assess specific long-term safety and efficacy measures. As such, it is critical that we re-evaluate current standards in pre-approval trials and the exposure of patients to a new drug prior to its approval [40].

Three-arm trials, including the experimental drug, an active reference treatment, and a placebo comparator, are recommended by the ICH and by the European Medicines Agency specifically for the clinical investigation of ADHD drugs. [20], [41] Three-arm trials serve two main purposes. First, the placebo comparator provides internal trial validation and demonstrates superiority of both drugs to the placebo control. Secondly, comparison of the new drug to an acknowledged standard yields comparative data on the safety and efficacy profiles of new drugs to other agents already available. We identified only 6 ADHD drugs that had been studied in comparison to another drug, indicating that ADHD drugs are introduced to the market with very little comparative data to guide clinicians on the new drug’s effectiveness in the context of all available options.

One of the limitations of this study is that, in some instances, the records from the FDA may have been incomplete. For each of the drugs, however, we were able to obtain the original approval packages. Given that many of these agents have been on the market for decades, there may be additional safety and efficacy information that has emerged in studies conducted after the original FDA approval. Nonetheless, our findings reflect the studies required in the FDA approval process, including the trials for a number of recently approved medications. Finally, our study was limited to ADHD drugs and additional study is needed to determine whether the FDA approval process is similar for other drug classes used in pediatric patients.

In conclusion, clinical trials conducted for the approval of many ADHD drugs have not been designed to assess rare adverse events or long-term safety and efficacy. Despite the large number of available drugs, comparative effectiveness trials are rare. While post-marketing studies can fill in some of the gaps, FDA enforcement is important to ensure that post-market surveillance studies are completed. An ambitious agenda to assess long-term outcomes in the millions of patients on these medications is warranted.
